# Multi-physics simulations and experimental comparisons for the optical and electrical forces acting on a silica nanoparticle trapped by a double-nanohole plasmonic nanopore sensor

**DOI:** 10.1016/j.sbsr.2023.100581

**Published:** 2023-08-11

**Authors:** Homayoun Asadzadeh, Scott Renkes, MinJun Kim, George Alexandrakis

**Affiliations:** a University of Texas at Arlington, Bioengineering Department, Arlington, TX 76010, USA; b Southern Methodist University, Department of Mechanical Engineering, Dallas, TX 75275, USA

**Keywords:** Solid-state nanopores, Double nanohole, Plasmonic, Optical trapping, Electrophoresis, Electroosmosis, Computational model, COMSOL

## Abstract

Bimodal optical-electrical data generated when a 20 nm diameter silica (SiO_2_) nanoparticle was trapped by a plasmonic nanopore sensor were simulated using Multiphysics COMSOL and compared with sensor measurements for closely matching experimental parameters. The nanosensor, employed self-induced back action (SIBA) to optically trap nanoparticles in the center of a double nanohole (DNH) structure on top a solid-state nanopores (ssNP). This SIBA actuated nanopore electrophoresis (SANE) sensor enables simultaneous capture of optical and electrical data generated by several underlying forces acting on the trapped SiO_2_ nanoparticle: plasmonic optical trapping, electroosmosis, electrophoresis, viscous drag, and heat conduction forces. The Multiphysics simulations enabled dissecting the relative contributions of those forces acting on the nanoparticle as a function of its location above and through the sensor’s ssNP. Comparisons between simulations and experiments demonstrated qualitative similarities in the optical and electrical time-series data generated as the nanoparticle entered and exited from the SANE sensor. These experimental parameter-matched simulations indicated that the competition between optical and electrical forces shifted the trapping equilibrium position close to the top opening of the ssNP, relative to the optical trapping force maximum that was located several nm above. The experimentally estimated minimum for the optical force needed to trap a SiO_2_ nanoparticle was consistent with corresponding simulation predictions of optical-electrical force balance. The comparison of Multiphysics simulations with experiments improves our understanding of the interplay between optical and electrical forces as a function of nanoparticle position across this plasmonic nanopore sensor.

## 1. Introduction

Solid state nanopores (ssNPs) enable label-free detection and analysis of biomolecules at the single-molecule level by use of resistive sensing of ionic current pulses. A voltage bias pushes analytes to move through the ssNP, causing a transient decrease in ionic current while this nanoscale aperture is blocked by translocating analyte [1–3]. The resulting time-series data of ionic current pulses has been used to detect DNA [4–7], proteins [8–14], miRNA [15–18], and other bioanalytes [19] has shown promise as a future, cheaper alternative for DNA sequencing [20,21].

Nanoaperture-focused plasmons in metallic films are a potentially enabling technology for directing analyte translocation via a nanopore, however this has been researched little to date. A self-induced back action (SIBA) mechanism can be used to achieve optical trapping in close proximity to metallic nanoapertures at low laser intensities [22]. In SIBA, a photon-scattering mediated force is triggered when a dielectric nanoparticle has a slightly different refractive index than the surrounding medium, in the vicinity of a plasmonic nanoaperture. Increased light transmission through the plasmonic nanoaperture as a consequence of the coupling of light to the far field via the dielectric nanoparticle allows for label-free sensing [23]. Gordon et al. demonstrated the use of double nanohole (DNH) nanoapertures as SIBA-mediated optical trapping for strong local field enhancement at the intersection of the nanoholes [24]. The Gordon group has published a number of research on the DNH structure’s design attributes [25–27] and its use in a variety of applications, such as the capture of nanoparticles [28–30] and individual protein molecules [24,31–33].

Several researchers have merged plasmonic optical nanosensing with nanopore sensing to better quantify bimolecular interactions. Researchers have suggested using light power to slow down the migration of molecules in order to improve the signal-to-noise ratio (SNR) of electrical nanopore signals, even though in some cases the additional optical measurements are only intended to enhance the diversity of data types collected synchronously [34]. Some methods include tweezing a DNA-tethered micrometer bead [35], with concurrent excitation of fluorescently labeled analytes for detection, to slow them down by adjusting the surface charge in conjunction with the consequences of electroosmotic flow [36]. In order to facilitate plasmonic augmentation of the optical field over the nanopore, bowtie-shaped nanoantennas [1,3,37–41] in gold (Au) were also developed. This led to concentrated heating because of higher ionic conductance, which did not reduce but rather increased the analyte translocation velocity, suggesting a compromise between throughput speed and measurement SNR.

We have originally described a plasmonic nanopore geometry consisting of a double nanohole (DNH) milled through an Au layer with an ssNP milled through its center [27]. The plasmon is focused between the DNH’s spart Au tips to enable SIBA-actuated nanopore electrophoresis (SANE), to trap analytes due to dielectric loading right above the nanopore entrance [42]. A key advantage of the SANE sensor is the enhanced specificity of analyte characterization by use of concurrent optical-electrical measurements [43].

The relative contributions of forces shaping the empirically observed optical trapping and nanopore translocation profiles are not, however, fully explained by these intriguing past findings. This is because the observed experimental data result from a competition between several forces, including plasmonic optical trapping, electroosmosis, electrophoresis, viscous drag, and heat conduction, all acting on an analyte simultaneously as it translocates through the SANE sensor. The purpose of this work was to perform detailed Multiphysics COMSOL simulations of the SANE sensor, with a 20 nm silica (SiO_2_) nanoparticle translocating through it, to disentangle the relative contributions of each of those forces as a function of nanoparticle position in the sensor. The simulations also identified an optical power threshold that needed to be reached for the SANE sensor to trap a nanoparticle. Where possible, experimental measurements were made to enable qualitative comparisons with simulations performed using matched physical parameters, to test for consistency and help gain insight into how the observed optical and electrical data were generated.

## 2. Materials and methods

### 2.1. Simulated sensor geometry and simulation parameters

**Fig. 1.**
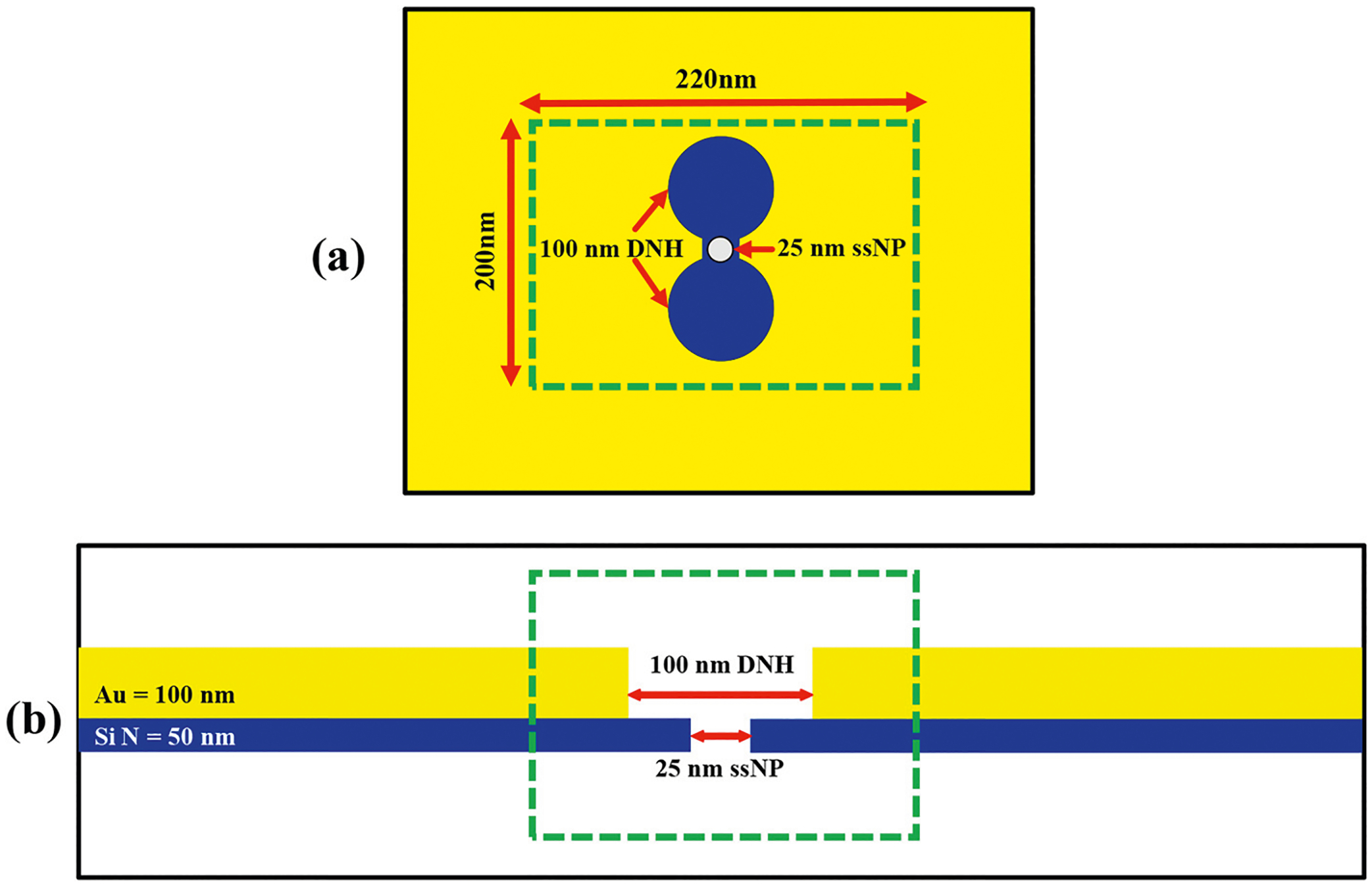
(a) Front side view and (b) cross-sectional view of the of SANE sensor geometry. The green-dashed rectangles indicate the boundaries of the COMSOL computational domain.

The simulated SANE sensor geometry is shown in Fig. 1, along with bounds of the area that was simulated (green dashed lines). The physical sensor, described in Section 2.4, was larger than the simulated area, but the regions not included in the simulations did not affect computational results.

The COMSOL simulations assumed the geometry of a DNH formed in Au, drilled into the physical sensor by Ne ion focused ion beam (FIB) milling (CNMS, Oak Ridge National Laboratory, Oak Ridge, TN). FIB endpoint detection during milling enabled stopping the process when the underlying Si_x_ N_y_ layer was reached and a 25 nm pore was milled through the Si_x_N_y_ layer, at the center of the DNH, with He ion FIB. The Au- Si_x_ N_y_ layers at the center of the sensor area were suspended as a bridge prior to FIB milling through successive chemical etching steps that removed the underlying material layers one by one, while front-side and back-side photomasks guided the precise alignment of the etching patterns, as described previously [4].

Only the central area of the sensor geometry, delineated by a green dashed line in Fig. 1(c) was included in the COMSOL simulations performed in this work. All other areas were assumed to be too far to affect computational results.

Table 1 lists the SANE sensor layer geometry thicknesses and the optical and electrical properties of materials in each sensor layer assumed in the simulations. The interface between the Au and Si_x_ N_y_ layers served as the axis origin (Nanoparticle Location = 0 nm). For all simulations, we assumed that the charge density of non-stoichiometric Si_x_ N_y_ is the same as the well-characterized stoichiometric Si_x_ N_y_.

Table 2 lists all relevant physical parameters assumed for the external laser illumination, voltage bias and analytes, reflecting the same parameters used in the physical experiments for 20 nm SiO_2_ nanoparticles, as described previously [44]. COMSOL simulations were performed for a 20 nm SiOS_2_ nanoparticle translocating through the midline of the SANE sensor. In order to compute the ionic current, an integral was taken over all-ion current flow which is passing along the DNH/ssNP [green dashed lines delineating a rectangular volume, Figs. 1 (a) and 1(b)], and for computing the optical transmission all angles of the light were collected and computed bellow the ssNP.

### 2.2. Governing equations for the optical-electrical fields in the simulated volume of the SANE sensor

The physics of ionic liquids can be approximated by three coupled classical equations: the Poisson equation relevant to electrostatics, the Nernst-Planck equation (NPE) describing ionic-flux, and the Navier-Stokes equation (NSE) governing the fluid flow. The Poisson eq. (PE), which implement the electrostatic field in the Au and Si_x_ N_y_ layers of the sensor, relates the electric potential V to the charge distribution ρv, and is given by:

(2-1) $$\nabla^{2}V=-\frac{\rho v}{\epsilon_{0}\epsilon_{r}}$$

where $\epsilon_{0}\sim 8.85\text{e}-12\;\text{F}/\text{m}$ is the permittivity of free space and $\epsilon_{r}$ is the relative permittivity, inherent property of the material and ρv is volume charge density. The charge distribution (volume charge density) can relate the electrostatic field with the ionic concentration and space charge density which will be effective in forming the electric Debye layer (EDL) on the nanopore’s wall in the SANE sensor. The related charge density equation can be represented in terms of the ionic concentrations as:

(2-2) $$\rho v=N_{A}e\sum z_{i}c_{i}$$

where $N_{A}\sim 6\times 1023{\;mol}^{-1}$ is the Avogadro’s constant, e~1.6e-19C represented the elementary charge, $z_{i}$ is the valence number and $c_{i}$ is the molar concentration of species i in the electrolyte. Monovalent electrolytes have been used in the majority of nanopore-based studies [44,56]. In the present study, 1 M Potassium Chloride (KCl) has been assumed as the electrolyte and the pH was set at 7.4.

The space charge density of this salt, because of its binary and monovalent nature, can be expressed as:

(2-3) $$\rho v=N_{A}e\left[C_{k+}-C_{Cl-}\right]$$

The flux J_i_ for each ionic species i is calculated using the Nernst-Planck equation:

(2-4) $${\text{J}}_{\text{i}}=-D_{i}\nabla_{ci}-Z\left(\frac{D_{i}}{RT}\right)FC_{i}\nabla V+uc_{i}$$

Looking at the equation closely, it may be deduced that the overall ionic flux is influenced by three components. The first part is caused by a concentration gradient, as described by Fick’s first law of diffusion [57]. The ionic flux generated by the formation of an electric field is the second component, and the advection of ionic species by the fluid velocity field is the third component. The first and third components are the most important contributors to ionic flux in the case of the SANE sensor because they cause the diffusion and convection of the ionic species in the electrolyte. Both of these components are coupled to the second component of electrokinetic flow (migration in electric field) and the related computational module in the COMSOL will be discussed in the next section.

The continuity and momentum (Navier-Stokes) equations can be used to define a Newtonian fluid in an isothermal condition and by coupling these equations with the computational fluid dynamic (CFD) technique in the entire computational domain of the SANE sensor. For simulating fluid motion, the Reynolds number Re=ρvL/μ needs to be computed. This is a dimensionless quantity which distinguishes the laminar from turbulent flow regime, where v and L are the flow velocity and length scale of the nanopore in the sensor (~160nm). In the present study, since fluid velocity is very low, Re is estimated to be ~0.0001, placing these simulations is in the laminar flow regime. Also, because the advective term in the Navier-Stokes equation can be neglected when viscous forces are greater than inertial forces, which are negligible here, and under steady-state conditions for fluid flow, we have a simplified momentum equation valid for low Re values, known as Creeping flow [47]:

(2-5) $$\nabla^{2}u=-\nabla P$$

The electrokinetic transport inside the nanopores is governed by the combination of Eqs. (2-1), (2-4), and (2-5).

To compute the light field distribution created by light focused onto the Maxwell’s eqs. (ME) is solved, given by:

(2-6) $$\nabla \times \mu_{r}^{-1}(\nabla \times E)-{k_{0}}^{2}\left(\varepsilon_{r}-\frac{j\sigma }{\omega \varepsilon_{0}}\right)E=0$$

where E is the electric field amplitude, $\varepsilon_{0}$ the permittivity of vacuum, j the current density, ω the angular frequency, σ the electrical conductivity, $\mu_{r}$ the relative permeability of the material and $\varepsilon_{r}$ is the relative permittivity.

The final equation is the convection heat transfer which is derived from energy equation and for the steady-state domain and can be represented by the heat eq. (HE):

(2-7) $$\rho {∁}_{p}\left(\frac{\partial T}{\partial t}+u.\nabla T\right)+(\nabla q)=\nabla u+Q$$

where ρ is the fluid density, ${∁}_{p}$ is the specific thermal capacity, u is the fluid velocity, q is the heat flux by conduction and Q is the heat source. Combination of Eqs. (2-6) and (2-7) will govern the temperature field created at the sensor by light beam illumination and absorption.

### 2.3. Forces on a dielectric nanoparticle

#### 2.3.1. Drag force

Under low *Re* flow regimes (creeping flow), the viscous drag force on a spherical target (the nanoparticle in our studies) can be expressed as:

(2-8) $$F_{Di}=-6\pi \eta rv$$

where η is viscosity of the medium, r the effective radius of the particle and v is the velocity of motion in the fluid.

#### 2.3.2. Electrophoretic force

Having the net electric field (Eq. (3–1)), the electrostatic force exerted on a charged ion particle is computed by [48]:

(2-9) $$F=ez_{p}E_{i}$$

#### 2.3.3. Dielectrophoretic force

The target particle can become polarized and induce a dipole moment in the presence of a spatially non-homogeneous electric field. As a result, a suspended particle can be pushed under such a field regardless of its surface charge by the Dielectrophoretic (DEP) force [39]:

(2-10) $$F_{DEP}=2\pi r^{3}\varepsilon_{m}\alpha \nabla E^{2}$$

where r is the particle radius, $\varepsilon_{m}$ the medium permittivity, and α is the Clausius-Mossotti factor represented by:

(2-11) $$\alpha =\frac{\sigma_{p}-\sigma_{m}}{\sigma_{p}+2\sigma_{m}}$$

where $\sigma_{p}$ and $\sigma_{m}$ are the complex permittivity of the particle and the medium, respectively. The DEP force exerted on a particle will push it towards either the stronger or the weaker field areas, depending on whether the sign is positive or negative. Since in the present study the α is equal to +0.5, the DEP force always pushes the nanoparticle to the region with stronger electric field, from *cis* to *trans*.

#### 2.3.4. Electroosmotic force

The electrolyte cationic species will move closer to the pore surface if there is a net negative surface charge density (σ) in the nanopore wall and an external electric field is applied. This positive-charge heavy surface suspended in the electrolyte gives boost to fluid flow. As soon as the voltage bias is applied, the cations are drawn towards the cathode (negatively charged electrode). This retarding force manifests as a retarding hydrodynamic force, which will add to the intrinsic viscosity of the fluid. This is known as the Electroosmotic force $\left(F_{\text{EOF}}\right)$ and is a function of Debye length emerging away from the charged pore wall surface. Because of the double layer developed on the pore surface, the electroosmotic mobility of the fluid may be reported as a function of the zeta potential, which can be calculated using Eq. (2-12):

(2-12) $$\mu_{\text{EOF}}=-\left(\frac{\varepsilon \zeta_{\text{pore}}}{\eta }\right)$$

where ε is the relative permittivity of the medium and ζ is the zeta-potential of the walls of the nanopore. Graham’s equation can be applied to approximate the zeta-potential in the above equation [40], which relates ζ to the estimated surface charge density of the nanopore. For the present work it was evaluated to be −0.02C/m^2^ [39].

Using the Helmholtz-Smoluchowski equation, the electroosmotic velocity can be represented as:

(2-13) $$u_{EOF}=\left(E.\mu_{\text{EOF}}\right)$$

Finally, the electroosmotic force ($F_{\text{EOF}}$) evaluated using Eq. (2-14):

(2-14) $$F_{\text{EOF}\;}=m\cdot \frac{d\left(u_{\text{EOF}}\right)}{dt}$$

where m represented the fluid mass in each grid of computational domain’s finite element meshes.

#### 2.3.5. Thermophoretic force

The thermophoretic force is exerted on a particle as a result of temperature variations in the background fluid. The thermophoretic force is defined as:

(2-15) $$F_{tp}=\frac{6\pi d_{p}\mu^{2}C_{s}\Lambda \nabla T}{\rho (2\Lambda +1)T}$$

where k is the thermal conductivity of the fluid, $k_{p}$ is the particle thermal conductivity, T the fluid temperature, and $C_{s}$ is a dimensionless constant equal to 1.17 and $\Lambda =k_{f}/k_{p}$ which represent the thermal conductivity of the fluid and nanoparticle, respectively [49].

#### 2.3.6. Light force

The potential energy of the particle in the optical trap created by the concentration of plasmonic field intensity at the center of the DNH is defined by:

(2-16) $$U=\frac{1}{2}\alpha \left|E^{2}\right|$$

where E represents the light field amplitude and α is the real part of complex permittivity, represented by:

(2-17) $$\alpha =\frac{{\varepsilon^{*}}_{p}-{\varepsilon^{*}}_{m}}{{\varepsilon^{*}}_{p}+2{\varepsilon^{*}}_{m}}$$

where ${\varepsilon^{*}}_{p}$ and ${\varepsilon^{*}}_{m}$ are the complex permittivity of the particle and the medium, respectively. The complex permittivity is expressed by $\varepsilon^{*}=\varepsilon -(j\sigma /\omega )$, where ε is the real permittivity, σ the conductivity, and ω is the angular frequency of the applied electric field. For a 20 nm SiO_2_ dielectric nanoparticle, the imaginary part of the permittivity is negligible, and the real part of the permittivity is ~1.1.

Then the light force can be defined as [50]:

(2-18) $$F=-\frac{dU}{dt}$$

#### 2.3.7. Brownian force

Collisions of continuous-phase fluid molecules with a particle produce the Brownian force on that particle. In submicron dimensions, Brownian motion of particles can become important. The Brownian force is treated as a Gaussian white noise random process with spectral intensity along all computational domains in this study, and can be represented as follows:

(2-19) $$F=\xi \sqrt{\frac{12\pi \mu Tr_{p}k_{B}}{\Delta t}}$$

where k_B_ = 1.3806488 e-23 J/K is the Boltzmann constant, r_p_ is the particle radius, Δt the time step that used by the solver, μ the fluid dynamic viscosity, T the absolute fluid temperature and ξ is a normally distributed random number with a mean of zero and unit standard deviation.

### 2.4. Finite element modeling of electrical-optical force fields in COMSOL

The impact of the above-described forces and their interactions on a 20 nm SiO_2_ nanoparticle was simulated by COMSOL Multiphysics (version 5.6, Natick, MA), approximating as closely as possible the parameters of physical experiments. The 2 D simulation domain was composed of two reservoirs of ionic liquid with two connecting structures in-between (DNH gap in the Au layer and the ssNP in the Si_x_ N_y_ layer), both 25 nm across, as shown in Fig. 1(a).

The simulation began with the NPE computing the dynamics of ions in the electrolyte, attained by the transport of diluted species module. The PE was then used to describe the electric field distribution throughout the simulation volume using the electrostatics module. The NSE was used to define the movement of water, realized by the laminar flow module. The ME was used to describe the light field distribution, attained by the wave optic module and the HE computed the temperature field using the heat transfer module.

**Fig. 2.**
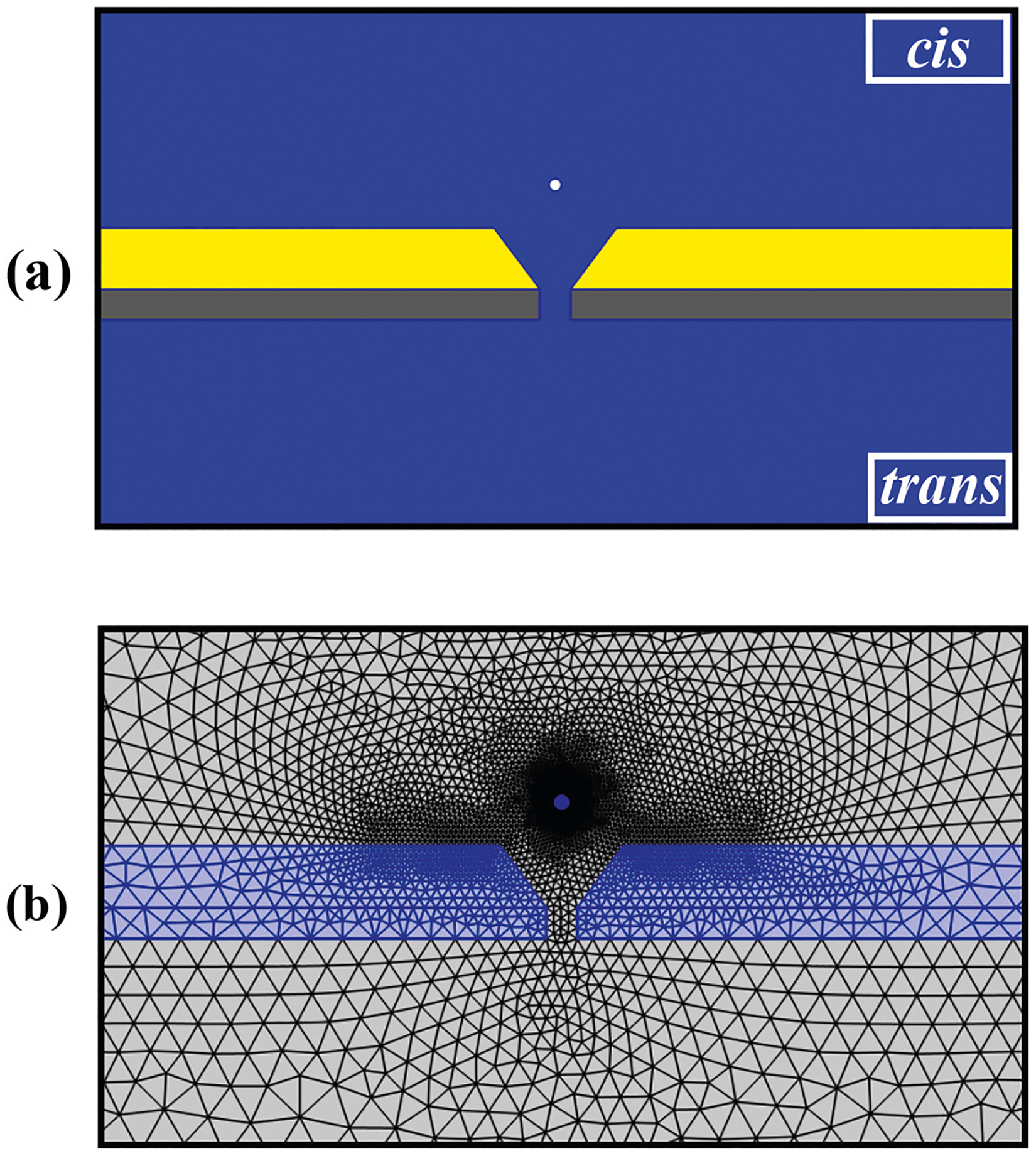
The computational domain of the SANE sensor is depicted. The yellow, gray, blue and green indicates the Au layer with the DNH gap, the Si_x_ N_y_ layer with the ssNP, the electrolyte and nanoparticle, respectively. (b) The mesh elements in the computational domain span a broad range of sizes with a finer mesh around the nanoparticle, sharp corners and around the nanopore volume.

In Fig. 2 the electrolyte domains (blue) the NPE, PE, and NSE were fully coupled and applied in a self-consistent way. In the Si_x_ N_y_ domain (gray), representing the nanopore membrane, the PE was applied to describe the electric field distribution and was coupled to the NPE and NSE. In the Au domain (yellow) the ME and HE were fully coupled. Also, for computing the forces on the nanoparticle the NPE, HE, NSE and PE were fully coupled.

For the boundary condition, in the electrostatic module for solving the PE, a surface charge density for the nanopore wall and nanoparticle surface were created (Table 2) and an electrical potential and ground was assigned at the top and bottom borders respectively (*cis* and *trans*). Also, the space charge density was assigned for the KCl domain. In the laminar flow module, the normal flow (P=0 Pa) was considered for the top and bottom surfaces as a boundary condition in solving the NSE [47]. On all other solid boundaries, a no-slip velocity boundary condition was adopted for solving the NSE. The electroosmotic velocity boundary condition was applied for the nanopore wall in order to compute the electroosmotic velocity field. A volume force was created by ‘space charge’ ×’electric field’ assigned to the KCl domains. For the transport of dilute species module, the initial concentrations on the boundaries of 150 mM KCl for the *trans* and *cis* sides were assigned to solve the NPEs, and zero (normal) electromigration and diffusion flux at all other solid boundaries [50]. Also, the ‘Convection’ and ‘*Migration in Electric Field’* were selected for the transport mechanisms in the laminar flow module. The diffusion coefficient and the concentration of each ion, i.e., K+, Cl−, were created and assigned in the transport of diluted species module (Table 2).

In the ‘*Wave optic’* module for solving the ME, a perfectly matched refractive index layer was used at the top and bottom surfaces to avoid back-scattering at the outer boundaries and perfect electrical conductor was assumed for side boundaries of the Au layer. For heat transfer module to compute the HE, thermal convection in all surrounded boundaries and thermal sources around the Au layer were applied as boundary conditions. Quadratic triangular elements were used in the finite element calculations.

Because the precision of numerical solutions is highly influenced by mesh size, a refined mesh was required in the region near the surface where the dependent variables gradients are prominent [Fig. 2(b)]. In this study, ‘Physics Controlled Mesh’ was chosen with the size ‘Finer’ in the mesh generation part of software to capture the small variations in potential, ionic concentration, and velocity near the charged membrane surface. It should be noted that the maximum element size far from the DNH was set to 1/10 of the light wavelength (830 nm) to solve the ME. Finally, the stationary study for solving the NPE, HE, NSE, and PE and a frequency domain study for solving the ME were assigned.

One limitation of the COMSOL package was that, in the present study one cannot couple the wave optic and ‘*Transport* of *dilute species*’ so the ME-calculated optical force on the nanoparticle was not coupled to all other forces. Therefore, it was not possible to let the nanoparticle propagate through the nanopore against the optical trapping force as part of a time-dependent simulation. Instead, the resultant of all forces was computed for different vertical positions along the midline of the nanopore and then by using the Newton’s second law and kinematic equations in Eq. (2-20) along the vertical path along the midline of the DNH/ssNP, the translocation time of the nanoparticle was computed:

(2-20) ∑F=m.a

### 2.5. SANE sensor fabrication

**Fig. 3.**
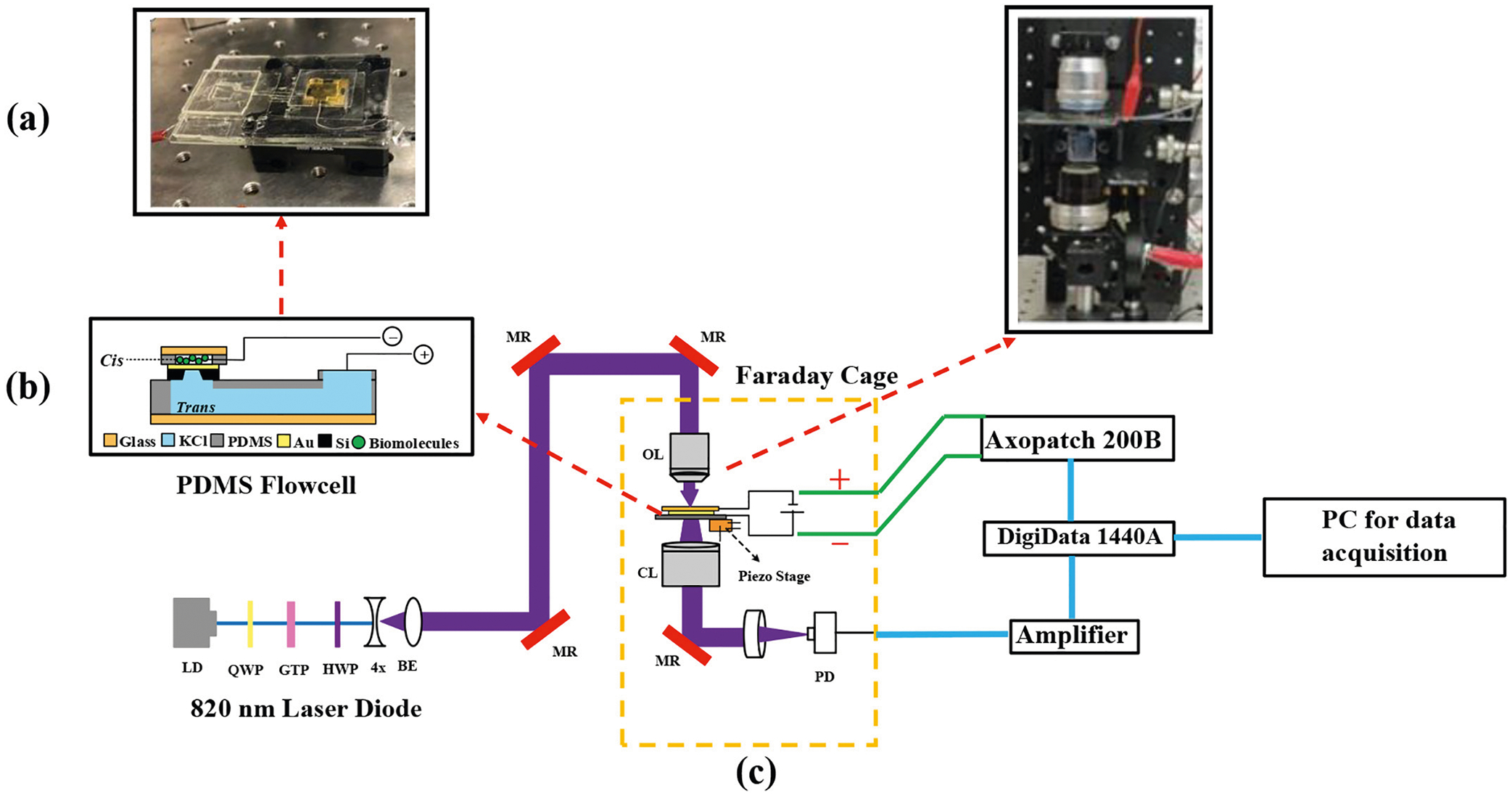
Complete experimental setup including flow cell, optical setup, and data acquisition equipment. (a) PDMS flow cell cross-sectional view with the SANE sensor. (b) Image of prepared PDMS flow cell with SANE chip ready for placement on piezo-controlled stage. (c) Complete optical setup with PDMS flow cell placement and measurement instruments. LD: laser diode, QWP: quarter wave plate, GTP: Glan-Thompson polarizer, HWP: half wave plate, 4× BE: 4× beam expander, MR: mirror, OL: Carl Zeiss 1.3 N.A. 63× objective lens, CL: condenser lens, PD: photodiode. The Chebyshev type-II filter was inserted in the setup just before data was digitized.

The procedure for fabricating the SANE sensor has previously been documented [51]. In summary, the sensors were created on clean 4-in. silicon (Si) wafers with a 500 nm silicon dioxide (SiO_2_) layer formed on top through thermal oxidation. A 60 nm layer of non-stoichiometric silicon nitride (Si_x_ N_y_) was then deposited using low-pressure chemical vapor deposition. On the wafer backside, a grid pattern was applied using a darkfield mask (positive photoresist S1813) to divide the wafer into individual chips measuring 15 mm × 15 mm. The mask also defined a square window of 786μm on each side, where the Si_x_ N_y_ layer was etched away using deep reactive ion etching with tertrafluoromethane (CF4) gas at a rate of 1 nm/min. Subsequently, the underlying SiO_2_ layer was etched using a 6:1 buffered hydrofluoric (BHF) acid until reaching the underlying Si layer. The backside was then subjected to an anisotropic etching process using a 22% tetramethylammonium hydroxide (TMAH) solution at 90 °C to create a 100μm window, leaving the overlying Si_x_ N_y_/SiO_2_ layers from the front side suspended. On the front side of the wafer, a 100 nm thick layer of gold (Au) with a 5 nm chromium (Cr) adhesion layer was deposited using the e-beam evaporation method at a rate of 0.1 nm/s. Alignment markers for focused ion beam (FIB) milling were patterned on this Au layer using photolithography, and the Au and Cr layers over the marker positions were etched using specific wet etchants. A thick layer of photoresist was applied as a protective coating, and then the wafer was diced into individual chips. Each chip underwent an acetone rinse to remove the photoresist layer, and the underlying SiO_2_ layer was eliminated using a 6:1 BHF solution. The individual chips were placed in a gas field ion source FIB (Carl Zeiss, ORION Nanofab, Peabody, MA), where the DNH nanostructures were milled through the Au layer using a Ne ion beam, and the nanopore was created in the Si_x_ N_y_ membrane at the center of the DNH structure [see Fig. 3(c)], using a He ion beam. The typical dimensions for the DNH structures utilized in this study were 100 nm diameter circles that intersected to create tapered edges with 15%−20% slope, converging towards a 25 nm diameter pore located in the middle of the structure.

### 2.6. Experimental setup

Fig. 3(c) depicts the experimental setup in schematic form. In brief, a laser diode (820 nm, L820P200, Thorlabs) emitted a collimated beam, which was transformed into a circularly polarized 2 mm diameter beam using an aspheric lens and a quarter-wave plate (QWP) (WPQ05M, Thorlabs). A Glan-Thompson linear polarizer (GTH10M, Thorlabs) and an adjustable half-wave plate (HWP) (WPH05M, Thorlabs) were utilized to select the optimal linear polarization aligned with the short axis of the DNH on each chip, enabling the optimal excitation of wedge plasmons.

The beam expander (4×, Newport) ensured that the back aperture of a 63× oil immersion objective lens (NA = 1.2, Zeiss C-Apochromat) was fully filled through a periscope. Precise positioning of the laser beam at the center of the DNH was achieved by employing the alignment markers and adjusting the controls of a piezo stage (MDT6938, Thorlabs) that held the chip. The sensor was enclosed within a transparent polydimethylsiloxane (PDMS) flow cell, fabricated according to our previous work [52], with a coverslip placed on top to focus light onto the center of the DNH on each chip. The transmitted light was collected using a condenser lens and subsequently directed onto a photodiode (PDA36A, Thorlabs). The PDMS chip holder [Fig. 3(b)] featured a *cis* chamber where protein solutions mixed with potassium chloride (KCl) were dispensed, while the *trans* side contained only KCl solution of the same molarity (150 mM). Two silver chloride-coated silver electrodes (Ag/AgCl), one in the *cis* chamber and the other in the *trans* chamber, were employed to apply a voltage bias across the nanopore at the center of the DNH structure. These electrodes were connected to an Axon Headstage (CV 203BU), which formed part of an Axon Axopatch 200B patch clamp amplifier and digitizer equipment (molecular devices) operating in voltage clamp mode. This setup enabled the measurement of changes in resistance caused by the flow of ionic current through the nanopore.

For each measurement session a baseline was established for the sensor utilized in the studies using 40 L of a 300 mM KCl solution with a pH of 7.4. The measurements were made using a command DC voltage that was maintained throughout all tests at 110 mV and 190 mV(− ve *cis* to + ve cis). Also, the recorded raw ionic current was subjected to a 20 Hz low-pass 8-pole Bessel filter using Axon Clampfit 10.6 software. This filtering process facilitated the examination of the effects of nanoparticle movement on the lower frequency range of the ionic current. To process the data, the event parameters from the binary file (.abf) generated by the pCLAMP program (Molecular Devices, San Jose, CA) were loaded into MATLAB (MathWorks, Natick, MA). All of the data presented in this study were collected using a single sensor to make comparisons between trials in this work as simple as feasible. The nanobead trapping rate data was compiled by measuring the time intervals between successive optical signal step-changes in time-series data. To assess statistical significance between trapping rates under different voltage bias a t-test was employed.

## 3. Results and discussion

**Fig. 4.**
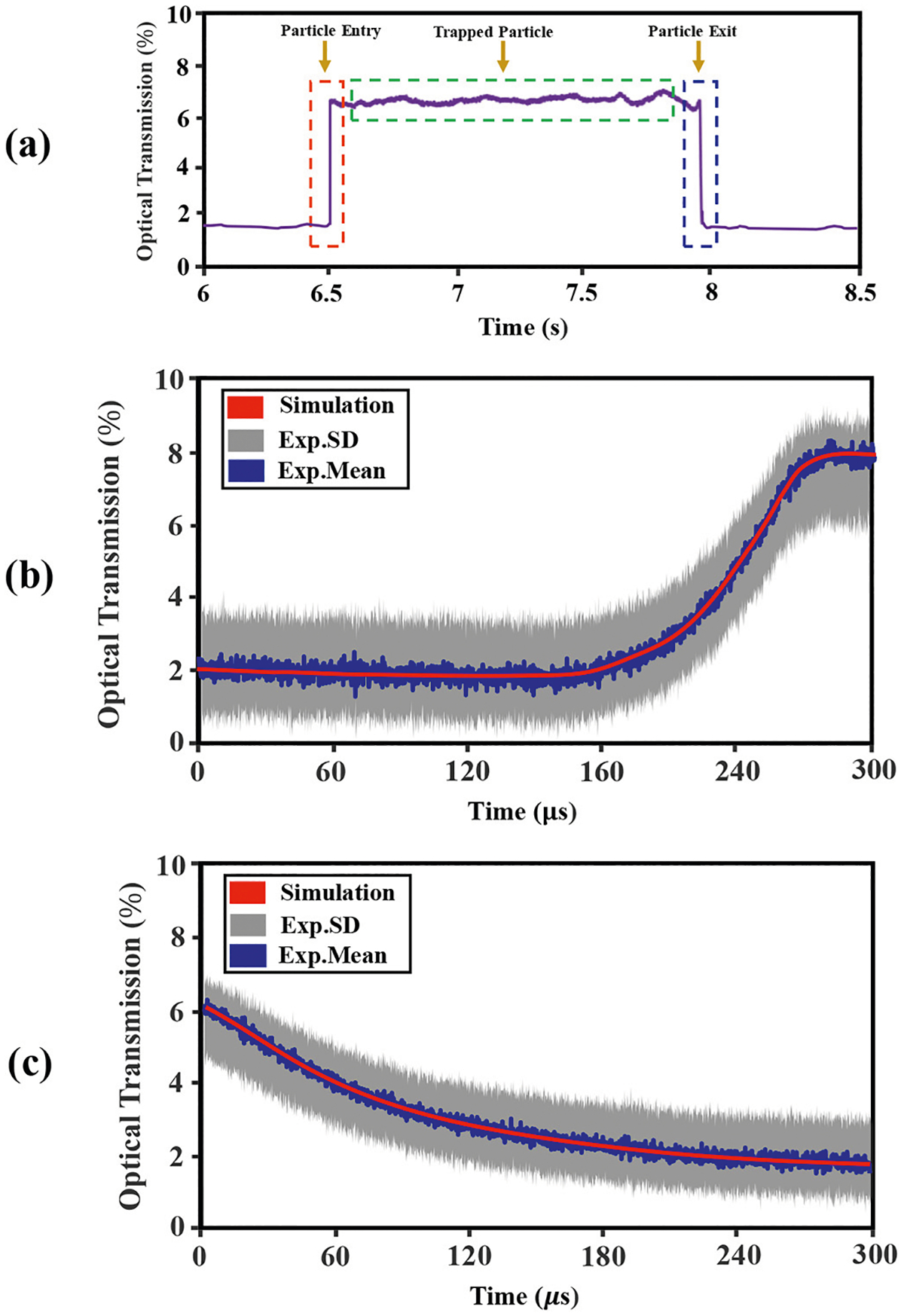
(a) Plot of experimentally measured optical transmission intensity versus time for the 20 nm SiO_2_ nanoparticle entering the optical trap (red dashed line), staying in the trap (green dashed line) and exiting the trap (blue dashed line) and time-magnified optical transmission change upon trap entrance (b) and exit (c). Computational results for the relative change in transmitted light intensity versus time upon trap entrance and exit ((c) and (c), red curves)) for the simulated SiO_2_ nanoparticle are compared against experimental results (mean percent transmission change, blue curves; standard deviation to the mean, gray curves).

A representative experimental observation of a multi-second trapping event by the SANE sensor for a 20 nm SiO_2_ nanoparticle is shown in Fig. 4(a). Fig. 4(b) shows a rise in transmitted light intensity during trapping and a decrease once the nanoparticle escapes the trap [Fig. 4 (c)].

**Fig. 5.**
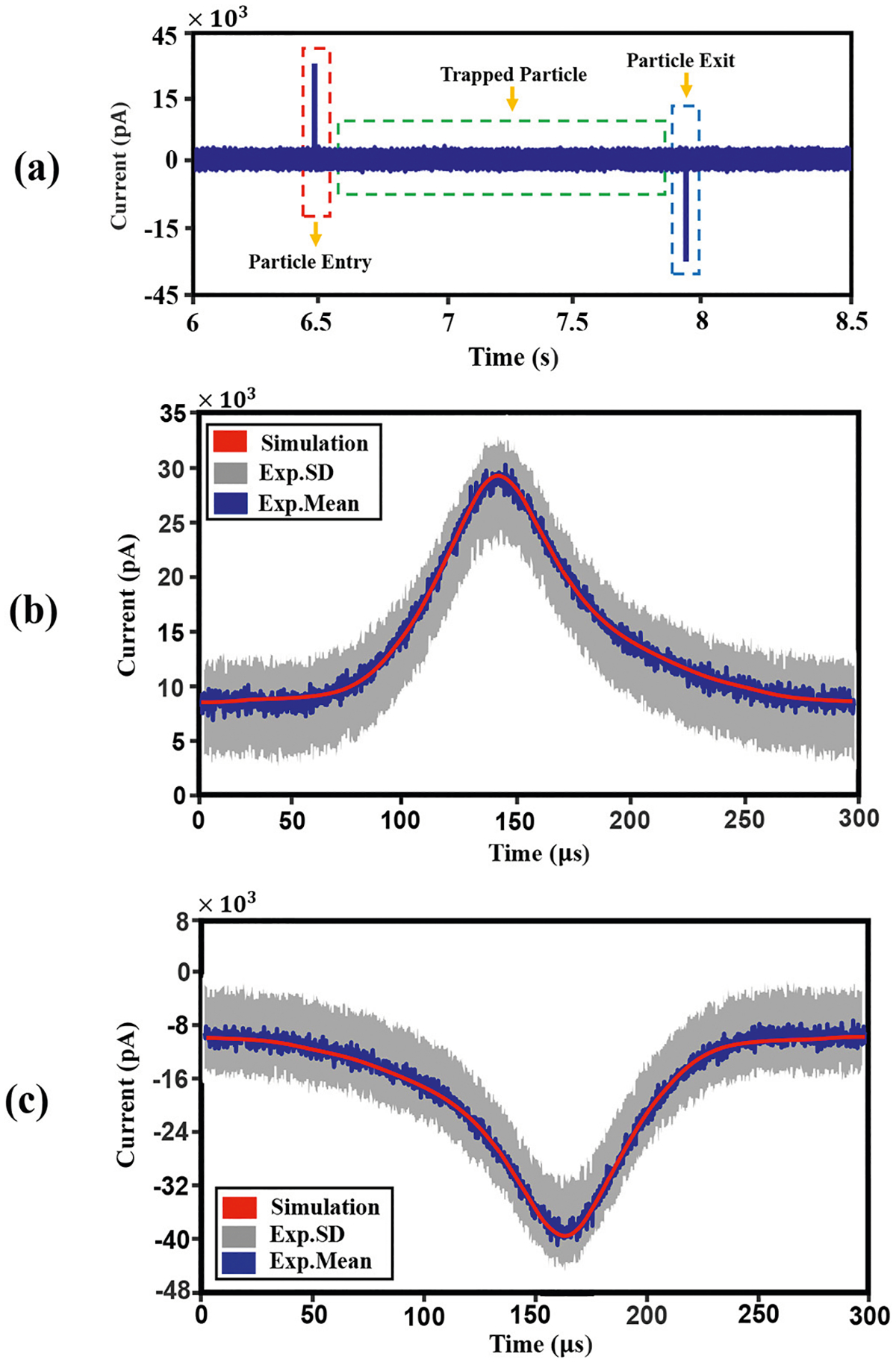
(a) Plot of experimentally measured electrical current versus time for the 20 nm SiO_2_ nanoparticle entering the optical trap (red dashed line), staying in the trap (green dashed line) and exiting the trap (blue dashed line) and time-magnified optical transmission change upon trap entrance (b) and exit (c). Magnified images of ionic current at particle entry (b) and exit (c) can be compared qualitatively related to experimental results with corresponding computational results (red curves) in (b) and (c).

The optical transmission through the DNH increased by about 10% as a result of the 20 nm nanoparticle’s dielectric loading of the trap [Particle entry; Fig. 4(a)]. Nanoparticle trap entry also created a high-frequency transient in the raw ionic current [Fig. 5(a)], with a maximum positive current estimated at 44 pA, which was roughly 19 times larger than the baseline nanopore current.

Upon nanoparticle escape, a distinctive negative ionic current pulse caused by transient blockade of the SANE sensor’s nanopore was detected as the nanoparticle moved past the optical trap, from the *cis* to the *trans* side of the desktop [Fig. 5(a), blue dotted line]. The optical transmission reduction event and this electrical spike happened simultaneously [Fig. 4(a), blue dashed line].

Experimental data and COMSOL simulations were compared in order to assess the validity of the computational approach to generate optical transmission and ionic current data. Figs. 4(b) and 4(c), show a magnified image in time for optical transmission during particle entry and particle exit, respectively. Optical transmission versus translocation time for various nanoparticle positions was calculated in simulation [Figs. 4(b) and 4(c), red curves] and was superimposed to the experimental data, showing good agreement [Figs. 4(b) and 4(c), 10μs time-bin mean value for current: blue noisy data; standard deviation: gray data].

Similarly, Figs. 5(b) and 5(c), show time-magnified images of electrical measurement during particle entry and particle exit, respectively. As in the case of optical translocations, the COMSOL computations showed good qualitative agreement with experimental data. Interestingly, the transient electrical signals during particle entry and exit were both ~310μs [Figs. 5(b) and 5(c)], as was the optical signal transient during exit [Fig. 4(c)]. However, the optical transient was significantly shorter [~120μs, Fig. 4(b)]. It is also noteworthy that the current change occurring upon trapping has positive directionality [Fig. 5(a)], indicating that current passes from *trans* to *cis*, whereas upon escaping the trap the current change is negative.

Conventional nanopores used for electrical sensing create spikes of only a single polarity, typically negative [42]. The dynamics driving these electrical spikes are discussed below, along with an explanation for the apparent slight asymmetry in transient electrical signals, both during optical trapping and the subsequent trap escape [Figs. 5(b) and 5(c)].

### 3.1. Computation of physical parameters driving the nanoparticle sensing process

**Fig. 6.**
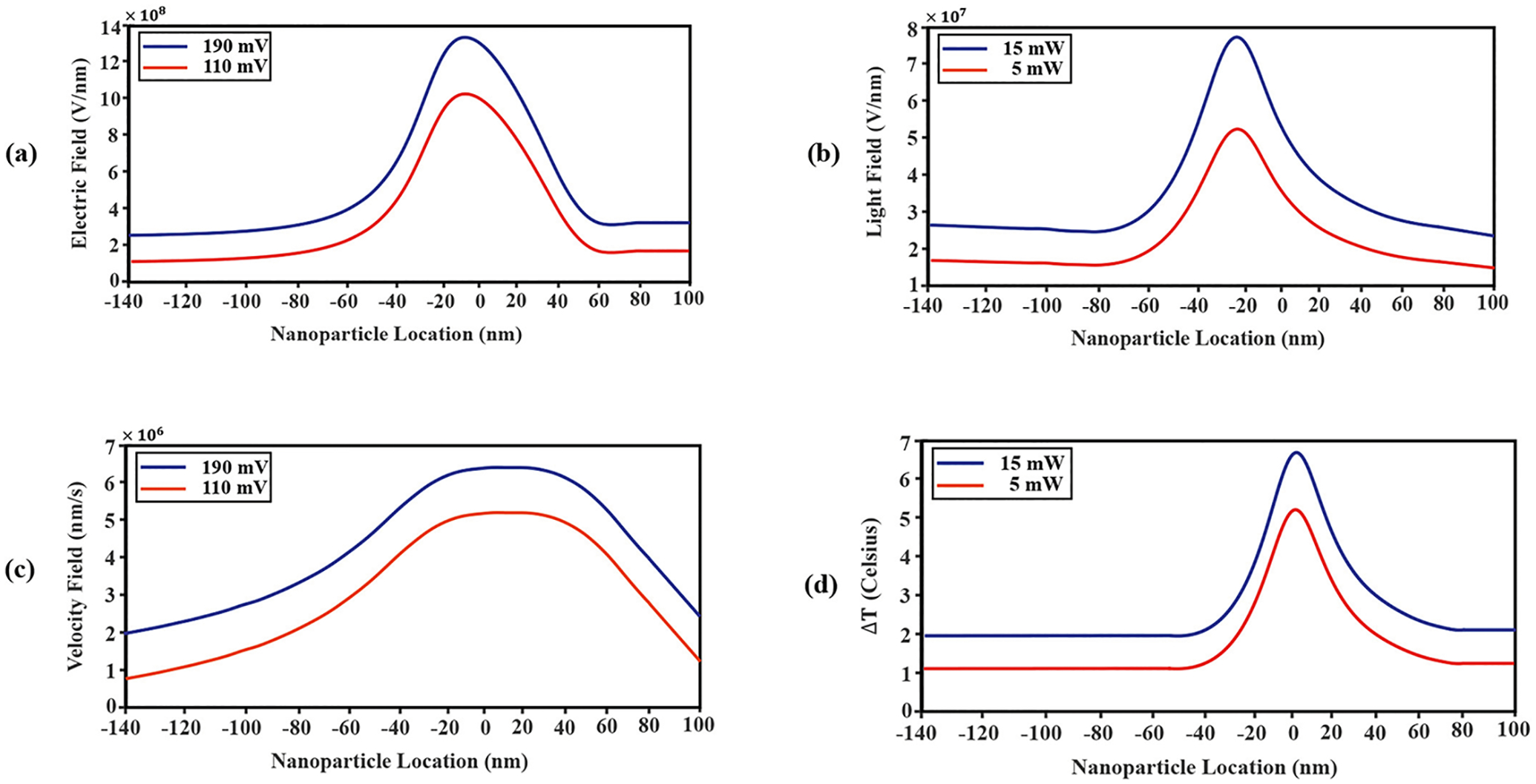
(a) Electrostatic field amplitude, (b) light field amplitude, (c) ionic fluid velocity field, (d) and temperature field for a nominal (110 mV) and the experimentally maximum possible (190 mV) external voltage bias values as a function of axial location across the sensor for the 20 nm SiO_2_ nanoparticle. The figure insets provide sample views of the spatial distribution of each of these parameters for a specific nanoparticle location.

Fig. 6(a) plots the axial electric field, originating from a distant electrode, as a function of nanoparticle location. Fig. 6(b) plots the light field intensity, originating from a focusing objective with matched specifications to the one used in experiments. Interestingly, both the electric and the light field reached their maxima at the same location of ~-9nm, slightly above the Au-Si_x_ N_y_ interface. Moreover, Fig. 6(a) shows that as voltage bias is increased, from a nominal experimental value (110 mV) to a voltage bias near the maximum that the Axopatch 200B system can operate under the relevant experimental conditions (190 mV), the corresponding electric field strength acting on the nanoparticle increases at all axial locations with no change in relative spatial shape. Similarly, Fig. 6(b) shows that as light intensity is increased, from a non-trapping to trapping-capable intensity (from 5 mW to 15 mW; determined experimentally), optical intensity increased, but its relative spatial profile was unchanged.

In contrast to the electric field bias and light field intensities that peaked just above the Au-Si_x_ N_y_ interface, the aqueous KCl fluid velocity and the fluid temperature peaked near ~0nm i.e., exactly at that interface [Fig. 6(c)]. Bernoulli’s principle led to the intuitive prediction that a surge in fluid velocity would occur at the ~0nm position when fluid rushed from the more open DNH area into the smaller nanopore channel. Also as expected, a higher applied voltage increased fluid velocity due to the increased electrophoretic force on KCl that dragged water molecules along with it. Lastly, the temperature field was also expected to have a maximum at the Au-Si_x_ N_y_ interface, as reproduced in the simulations [Fig. 6(d)], due to Joule heating being highest at the sharp tips of the DNH and the underlying Si_x_ N_y_ being a dielectric material that does not conduct heat at well as Au.

### 3.2. Computation of the forces of different physical origin acting on the nanoparticle with respect to its axial position along the sensor

#### 3.2.1. Forces pushing from cis to trans

**Fig. 7.**
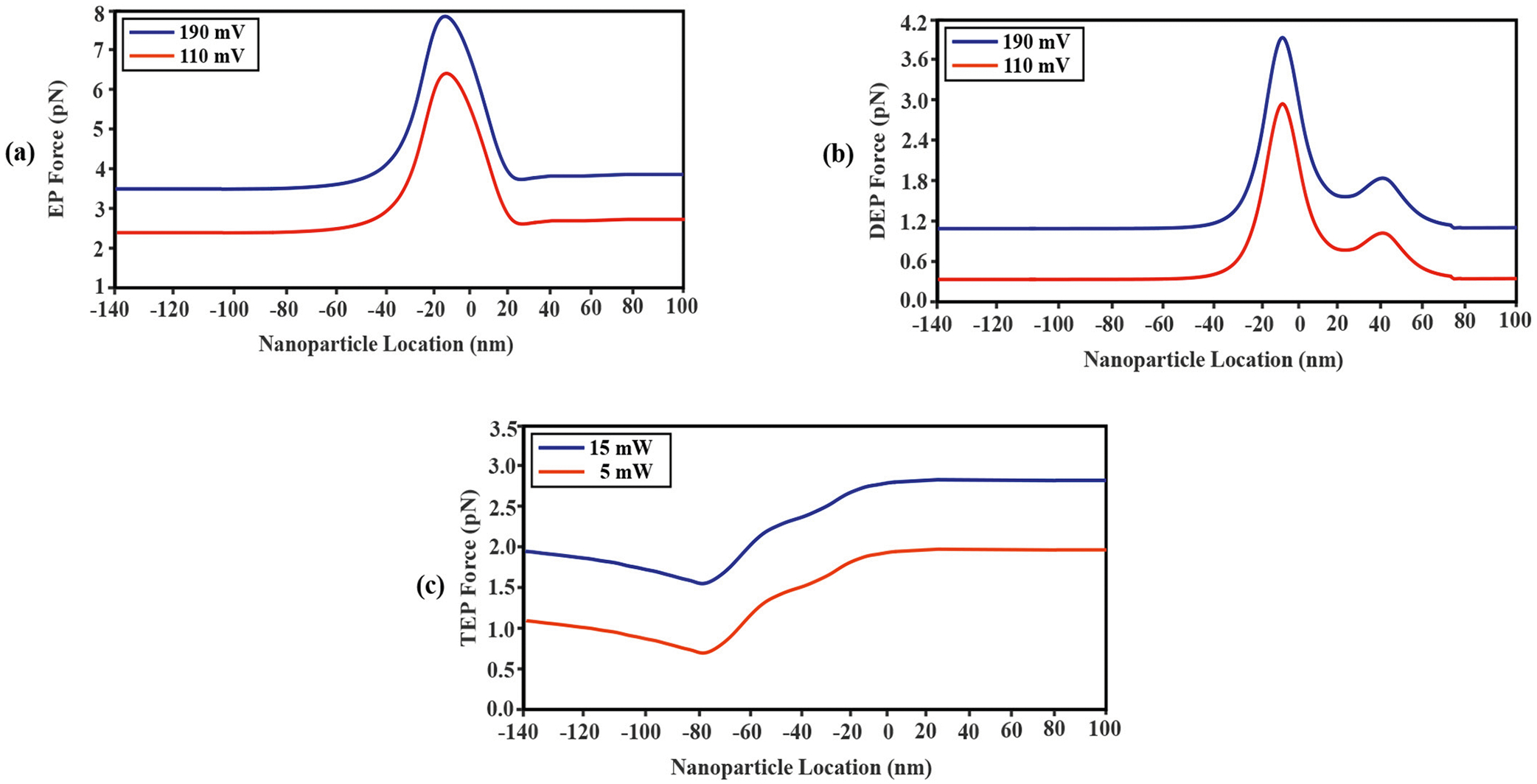
Plots of the electrophoretic (a), dielectrophoretic (b), and thermophoretic (c) forces as a function of nanoparticle axial location and voltage bias (negative polarity in cis).

The electrophoretic force acting on the nanoparticle was computed as a function of its axial position for two distinct external voltage bias levels, the nominal 110 mV and the maximum possible 190 mV in our experimental setup [Eq. (2-9); Fig. 7(a)]. The electrostatic field’s peak value (x=-9nm) coincided with the peak of the electrostatic field [~-9nm, Fig. 6(a)], as expected. Also, a rise in voltage bias level likewise enhances the strength of the electrophoretic force [Fig. 7(a), blue curve], pushing that nanoparticle from *cis* to *trans*. Furthermore, by solving Eq. (2-10), it is possible to determine the dielectrophoretic force acting on the nanoparticle as a function of the axial placement [Fig. 7 (b)]. Similarly, to the electrophoretic force, the peak of the dielectrophoretic force occurred just above the Au-Si_x_ N_y_ interface and the magnitude of this force increased with increasing voltage bias. It is important to note that while the dielectrophoretic force depends on the polarizability of the nanoparticle and the magnitude of the electric field gradient, the electrophoretic force depends on the electric field polarity and the electrical charge of the nanoparticle [37]. For the experimental conditions studied in this work, the negative charge of the particle (zeta potential = −40 mV) and the negative polarity of the field created a positive electric force. Similarly, the dielectrophoretic force was a product of positive quantities and therefore both forces push the nanoparticle from *cis* to *trans*. Because of the electric dielectric layer, positive electrical charge was present on the nanopore wall. As a result, the nanoparticle’s polarizability increased, which in turn raised the dielectrophoretic force. The thermophoretic force was finally determined for two different laser powers, one non-trapping and the other trapping capable, as a function of axial nanoparticle placement [Eq. (2-15)]. The computed thermophoretic force profiles followed the local temperature profiles [Fig. 6(d)]. This was as expected, as the Au-Si_x_ N_y_ interface was the axial location with highest intensity of plasmonically concentrated light intensity, creating heating [Fig. 7(c)]. Due to this sensor’s shape and illumination geometry, though particles had to overcome the backpush as they entered the optical trap, once there, they had a downhill-facing temperature gradient pushing the nanoparticle from *cis* to *trans*, just like the electrophoretic and dielectrophoretic forces.

#### 3.2.2. Forces pushing from trans to cis

**Fig. 8.**
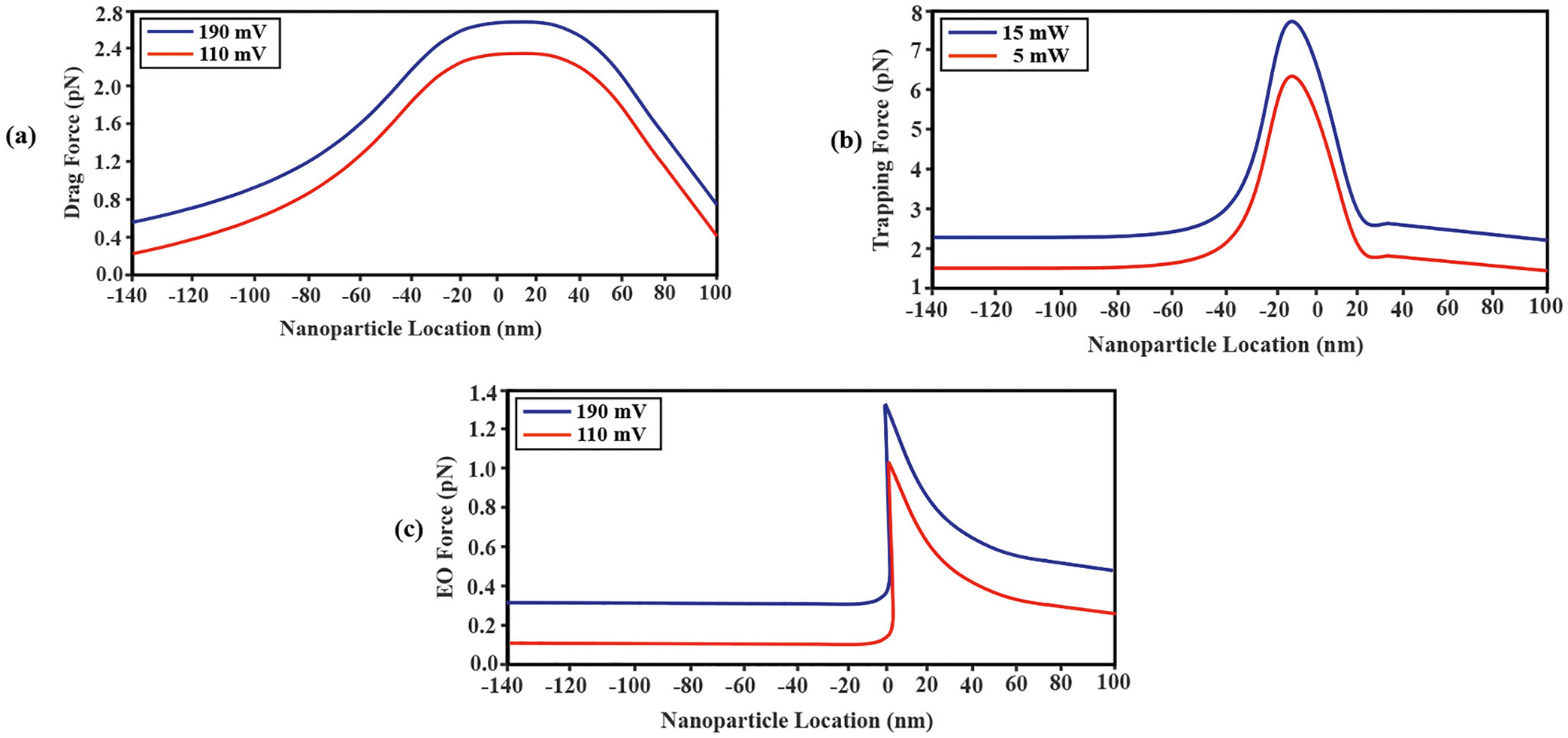
Plots of the drag (a), trapping (b), and electroosmotic (c) forces as a function of nanoparticle axial location, voltage bias (negative polarity in *cis*) and laser power.

One of the two forces slowing down molecular translocations was the viscous drag force, which was calculated for different nanoparticle axial positions for the same two external voltage biases as above [Eq. (2-8); Fig. 8(a)]. Where the electrostatic field and, thus, the velocity magnitude, are at their maximums, the drag force is greatest [49]. Because the drag force always opposes the velocity vector, it can be seen that the negatively charged nanoparticle was pushed from *trans* to *cis* by viscous drag. Moreover, the amplitude of this force grew as the voltage bias rose [37] [Fig. 8(a)]. Another force opposing nanoparticle translocation was the light-induced SIBA force, which was computed for each of the two laser powers, resulting in non-trapping and trapping experimentally, as a function of nanoparticle axial position [Fig. 8(b)], with the optical force increasing with laser power, as expected from Eq. (2-18).

Lastly, for the experimental parameters used in this work, the simulations showed that the electroosmotic force was also opposing nanoparticle translocation [Eq. (2-14); Fig. 8(c)]. The opposing force was created by cationic analyte flow from *trans* to *cis*, which was itself generated by the ionic charging of the nanopore wall. This positively charged surface that was suspended in the electrolyte caused a fluid flow that directed the fluid in the direction of the anode electrode. Crucially, it was assumed that the electroosmotic force did not exist in the DNH layer since, with Au being a conductor, its surface charge density would be zero and a Debye layer would not form.

**Fig. 9.**
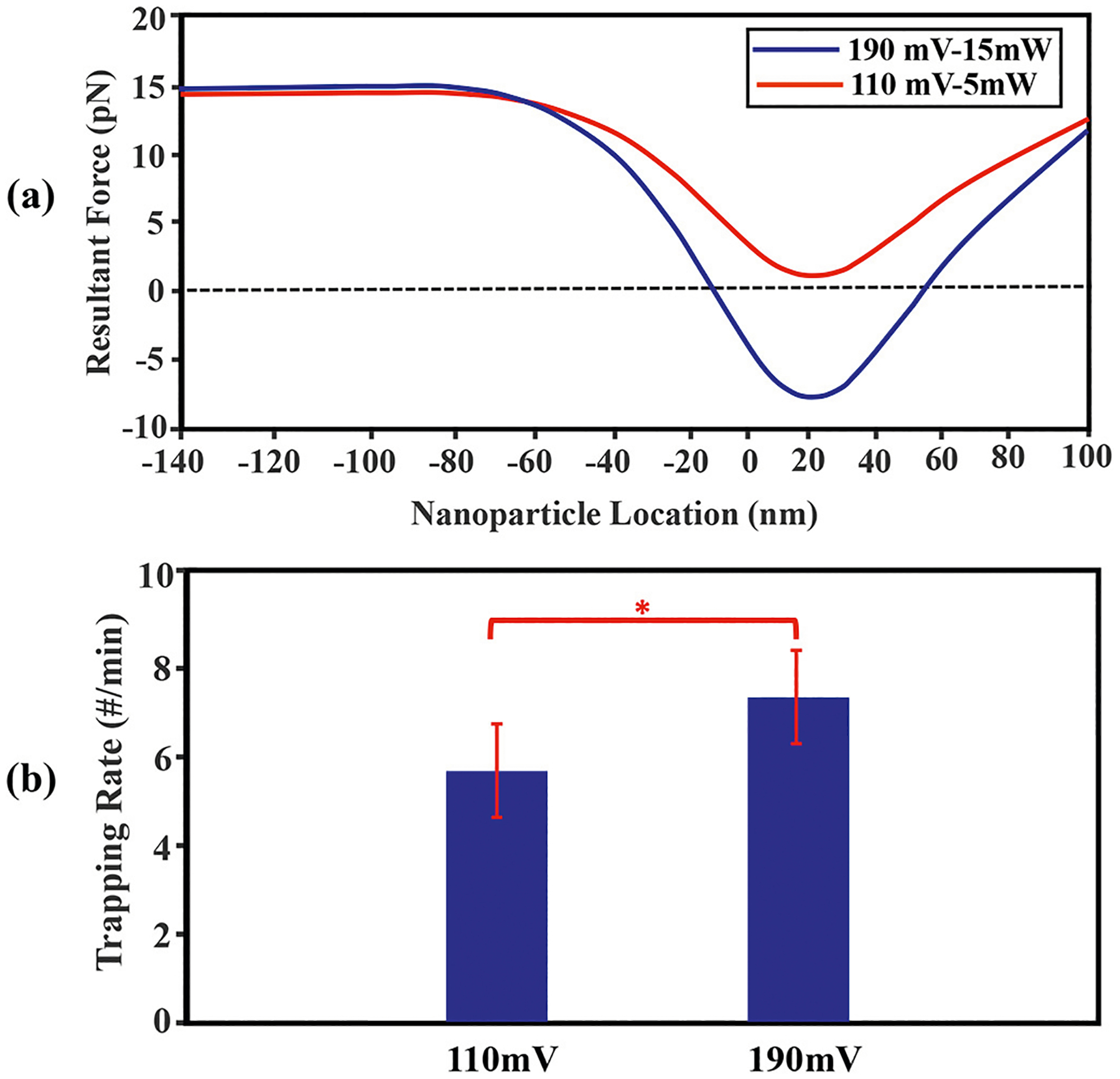
Plots of EP, DEP, TEP, EO, trapping and drag forces (a) forces in the SANE sensor, and EP, DEP, EO and as a function of nanoparticle axial locations in various voltage biases and laser powers and (b) plots of experimental results of the trapping event rate for the two different voltage biases at 15 mW (no trapping events were detected at 5 mW at any voltage bias).

Fig. 9 demonstrate the summation of all forces in the SANE sensor which already computed and showed in Figs. 7 and 8 for different laser powers and voltage biases. Fig. 7(a) indicates that for lower values of the laser power and voltage bias (blue curve) the resultant force is smaller in comparison with higher corresponding values (red curve). Fig. 7(b) represents the experimental results comparing nanoparticle trapping event rates at two different voltage biases. The results revealed that the trapping rate was statistically significantly higher for the 190 mV voltage bias compared to 110mV(p=0.04). This finding indicates that the application of a higher voltage bias significantly enhances the trapping capability of the system, as expected. To assess the statistical significance of these observations, error bars were incorporated into the diagram. The presence of statistically significant error bars supports the conclusion that the disparity in trapping rates between the two voltage bias values is not due to random variation but rather reflects a genuine difference. In summary, the bar diagram provides clear visual evidence that increasing the voltage bias to 190 mV leads to a substantially higher trapping rate compared to the 110 mV bias. The inclusion of statistically significant error bars adds further credibility to these findings, highlighting the robustness of the observed differences.

Table 3 lists the maximum values of all the COMSOL-simulated forces that are known to exert pressure on the nanoparticle inside the SANE sensor. The forces in this table are split into two groups: those that moved the nanoparticle from *trans* to *cis* (TR, EOF, and viscous drag) and those that pushed it from *cis* to *trans* (EP, DEP, and TEP). To match the experimental conditions, the EP, DEP, EOF, and viscous drag were computed for the 100 mV and 110 mV external voltage bias, and the TEP and TR forces were computed for the 5 mW and 15 mW laser powers. According to these computations, the Brownian force (Eq. (2-19)) was the smallest size force operating on the nanoparticle, while the EP was the strongest force acting on it.

The purpose of this work was to improve our current understanding of the relative contributions of the optical and electrical forces acting on a 20 nm SiO_2_ nanoparticle as it approached, was trapped and then escaped a type of plasmonic nanopore known as the SANE sensor. To that end, COMSOL Multiphysics simulations were employed to help estimate the magnitude and spatial variation of these forces as the nanoparticle translocated through the sensor. Given that it was not readily feasible to measure experimentally all of those forces and their spatial variations, the simulations were used to predict the optical and electrical time-series signal profiles generated due to the interactions of those underlying forces. Simulation results were then compared to the experimentally measured optical and electrical time-series signals generated simultaneously as the nanoparticle translocated through the sensor. Subsequent comparisons between simulations and experiments shed light onto how those interacting forces control how nanoparticles travel through the sensor, while also bringing to the surface some limitations of the COMSOL Multiphysics package.

Firstly, the step-changes in optical transmission and the simultaneous transient changes in current conduction through the ssNP, when the nanoparticle entered and exited the optical trap of the SANE sensor were simulated. When the nanoparticle entered the optical trap transmission increased due to increased dielectric loading, as described previously [31,43]. This step-increase in transmitted intensity [Fig. 4 (b)] lasted until the particle escaped the trap [Fig. 4(c)]. At the same time, as the nanoparticle approached the top side of the SANE sensor it displaced electrolyte-containing fluid, which created a transient negative charge deficit. The latter drove a transient current for *trans* to *cis* until charges equilibrated again [Fig. 5(b)]. When the nanoparticle escaped from the optical trap, it blocked the ssNP transiently, while translocating through it, creating a negative current spike [Fig. 5(c)]. Therefore, in contrast to the more common dielectric ssNP that only present a negative current spike during translocation, the SANE sensor data show bipolar spikes for each translocation event.

A practical limitation encountered on the computational side of this work was that although all the equations describing the influence of the externally applied volage bias on to the sensor, the KCl ionic liquid, and the dielectric nanoparticle could be solved fully coupled in COMSOL Multiphysics, this software package did not offer coupling with Maxwell’s equations. Therefore, the optical forces and the heating gradient near the regions where plasmonic focusing was highest, could not be coupled to the forces created by the voltage bias. An additional complication encountered was that the COMSOL converges in slower time steps than the rate of change of these physical phenomena because of numerical algorithms used for solving the underlying mathematical equations which can introduce complexities that lead to slow solver convergence. Also, transient effects with rapid changes may require smaller time steps to accurately capture the dynamics of the system. As a result of these software limitations, the time-dependent dynamics of the nanoparticle through the sensor could not be computed directly. Instead, in this work the optical transmission (Fig. 4) and electrical current conduction (Fig. 5) profiles were computed for a range of static axial positions of the nanoparticle throughout the sensor. A simple and approximate physical model involving Newton’ second law was implemented to compute the nanoparticle’s instantaneous velocities at different locations through the sensor, which were in turn used to map axial position to time. As a result of these computational limitations, the optical signal simulations could only be matched qualitatively to the experimentally measured profiles of relative optical signal change i.e., to within a multiplicative scaling factor [Figs. 4(b) and 4(c)]. Qualitatively similar profiles were also attained between computation and experiment for current spike amplitudes and translocation times. Interestingly, the simulations also reproduced the temporal asymmetry seen in experimentally measured current spikes [Figs. 5(b) – 5(c)]. It is interesting that the same type of asymmetry across time in Fig. 5(c) is seen across space in Fig. 9(a). This similarity is likely not coincidental and suggests a faster charging-discharging of the conical Au optical trap versus the slightly slower charging-discharging of the underlying cylindrical Si_x_ N_y_ nanopore as the nanoparticle translocates across the sensor.

In the next step, the simulations were used to compute the spatial variation of key physical parameters that determined the relative magnitude of forces acting on the nanoparticle as a function of axial position along the SANE sensor. The simulation results indicated that both the electric field due to the external voltage bias and the electric field of the light peaked above the DNH/ssNP interface [Figs. 6(a) and 6(b)]. Despite the qualitative similarity of these two profiles, these are likely explained but two distinct physical mechanisms. In the case of the applied voltage bias, the conductive Au layer created mirror charges that in turn created a near-zero equipotential surface on the *cis* Au surface. As a result, the highest value of the applied voltage, and its associated electric field amplitude, were maximum just above the Au surface and created a force that pushed from *cis* to *trans* for the experimental parameters used. On the other hand, in the case of the light electric field, the sharp corners of the Au material at the narrowest point of the DNH created strong plasmonic field focusing just above the mouth of the underlying ssNP. Plasmonic focusing is the key reason why nanoparticle trapping by the SANE sensor does not require a high-power laser source and yet the resulting optical force is large enough to oppose nanoparticle translocation.

The triangular shape of the DNH tips also affected indirectly the ionic fluid velocity profile through the sensor [Fig. 6(c)]. The localize heating of the DNH tips created a localized temperature gradient, starting just above the ssNP [Fig. 6(d)] that pushed the nanoparticle towards the ssNP by the resulting TEP force. Also, the local temperature profile around the DNH/ssNP was slightly asymmetric, presumably because of the axial asymmetry of the sensor geometry in the vicinity of the DNH that allowed for more rapid cooling from the top (conductive electrolyte) versus the bottom (Si_x_ N_y_ is a dielectric material) of the interface. Additionally, although no direct comparison with prior published work is available, our computational predictions of temperature rise in the vicinity of the DNH tips is consistent with prior studies. Specifically, Anyika et al. [53] have used a 973 nm laser at ~15mW to trap a 25 nm polystyrene sphere in a similar DNH geometry to this work, which resulted in a local temperature increase of up to 10 *C*°. Similarly, Jing et al. [53] reported a local temperature increase of $\sim 8C^{{}^{\circ}}$ for ~3mW of illumination at 1064 nm. It is important to note that both of these studies intentionally used longer laser wavelengths than the 820 nm used in this study, so as to induce local heating and increase convection-driven flow through the DNH. The slightly lower temperature increases with maxima in the $5-7\;C^{{}^{\circ}}$ range reported in this work are therefore consistent with these prior studies.

The EOP forces also contributed to enhancing forward fluid movement, and therefore forward nanoparticle movement, due to the additional current created in reaction to the counterion charges accumulated on the ssNP wall. Lastly, that presence of the ssNP below the DNH narrowed the volume through which fluid could travel, creating a Bernoulli effect that increased ionic fluid velocity driven by electrophoresis at zero pressure differential between the *cis* and *trans* reservoirs. In all, the results in Fig. 7 show that optical and electrical forces pushing the nanoparticle towards or away from the sensor were of comparable magnitudes, and it was their vectorial sum along the axial direction that determined where and under what conditions the nanoparticle was trapped.

Simulation results were then grouped by forces promoting nanoparticle translocation versus ones opposing it. Of the forces promoting translocation, electrophoresis was the strongest. Comparison of Fig. 6(a) to Fig. 7(a) suggests that the EP force’s relative strength was highest at the location where the external voltage bias was maximum. DEP forces also existed due to electric field gradients. For the SANE sensor geometry, the steepest electric field gradients and therefore the DEP force magnitude occurred just before the entrance and just before the exit (secondary peak) of the ssNP [Fig. 7(a)]. The TEP force was understandably broader than the temperature profile, as heat diffusion and convection tended to reduce the steepness of the temperature gradient created near the sharp corners of the DNH. Lastly, the electroosmotic force was the smallest of the opposing forces and was considered to be non-zero over the walls of the ssNP, as the Au material of the DNH above it was considered to be an ideal conductor. In practice, the Au surface could also form a Debye layer by pH -dependent proton adsorption of oxide species from solution [54,55].

Of the forces opposing translocation, the optical one was the strongest. Interestingly, the light wave interference patterns created a strong and spatially narrow force maximum near the DNH/ssNP interface, where the nanoparticle was likely trapped the longest, as well as secondary maxima on either side [Fig. 8(b)]. Lasty, the viscous drag opposition force [Fig. 8(a)], though smaller in amplitude than the optical force, had a more spatially extended axial profile that mirrored that of the ionic fluid velocity [Fig. 6(c)].

Fig. 9 presents the comprehensive summation of all computed forces, providing a visual representation of the intricate interplay and resultant effects observed across the length of the sensor. When laser power is increased from 5 mW to 15 mW, the resultant force threshold crosses over from positive to negative values, indicating that trapping occurs. The corresponding bar chart in Fig. 9(b) presents time-averaged nanoparticle trapping event rates over a data acquisition interval lasting 2 h. Our finding of no trapping at all at 5 mW and higher trapping rates at 15 mW with 190 mV bias (p=0.04) is consistent with the simulation results.

## 4. Conclusion

In summary, the simulations conducted in this study have provided valuable insights into the balance of multiple forces that control the behavior of the SANE plasmonic nanopore sensor. The results indicate that the dominant force near the sensor is the electrophoretic force, while the optical force reaches its maximum at the center of the sensor, as expected for effective trapping. The simulations also shed light on the relative contributions of various additional forces at different axial positions along the sensor, that could be altered e.g., by changing voltage bias or electrolyte concentration between sensor chambers, to control nanoparticle transport in future experiments.

While the simulations suggest that the combined effects of the optical force, electroosmotic force, and viscous drag are only slightly greater than the combined effects of the electrophoretic, dielectrophoretic, and thermophoretic forces, it is important to acknowledge that external perturbations could potentially disrupt the optical trapping. Brownian motion was found to have a minimal impact on the nanoparticle’s escape from the optical trap, but other factors present in physical experiments, such as laser power fluctuations and voltage bias fluctuations, may contribute to unintended trap escape. Additionally, the accumulation of multiple nanoparticles within the trapping volume, creating a sort of traffic jam immediately above a nanoparticle located at the center of the sensor, could lead to collisions and potential disruption of trapping events.

Furthermore, it is worth noting that while the presented results are approximate due to the limitations of the COMSOL solver in coupling Maxwell’s equations with the transport of diluted species and electrostatic modules, they still provide a qualitative understanding of the forces at play in the plasmonic nanopore sensor. These simulations can aid in the future interpretation of biomolecular signals obtained using this sensor and serve as a foundation for research aiming to refine the sensor’s performance for the control of biomolecular transport.

## Figures and Tables

**Table 1:** Optical and electrical properties assumed for the sensor’s Au and Si_x_ N_y_ layers.

Material	Thickness (nm)	Optical properties	Electrical Properties (Dielectric constant/Conductivity)
Gold	100	1.33	6.9 / 45.6 [S/m]
Si^x^ N^y^	50	1.8	9.5 / Negligible

**Table 2:** Physical and chemical parameters assumed in the COMSOL computations performed in this work.

Description	Expression
Surface charge density of Si_3_N_4_	−0.02 [C/m^2^]
Surface charge density of Au	0 [C/m^2^]
The concentration of KCl	1 [mol·dm^–3^]
K+ diffusion coefficient	1.96e–9 [m^2^·s^–1^]
Cl− diffusion coefficient	2.03e-9 [m^2^·s^–1^]
Valence of K +	1
Valence of CL −	−1
Laser power	15–5 [mW]
Voltage bias	110–190 [mV]
Temperature	300[K]
Relative Permittivity of Electrolyte	80
Permittivity of Vacuum	8.85 ×10^–12^ [F/m]
Viscosity of solution	10^–3^ [Pa. s]
pH	7.4
Wavelength	830 [nm]
Space charge density of KCL	96485e3 [C/mol]

**Table 3:** Maximum value and directionality of each of the forces known to act on a 20 nm SiO_2_ nanoparticle in the SANE sensor.

Type of Forces	Symbol	Direction	Voltage (mV) & Laser Power (mW)	Maximum Value (pN)
Electrophoretic	$F_{EP}$	*cis to trans*	110 mV / 190 mV	+4.01 / +4.55
Dielectrophoretic	$F_{DEP}$	*cis to trans*	110 mV / 190 mV	+1.69 / +1.09
Thermophoretic	$F_{TEP}$	*cis to trans*	5 mW/15 mW	+1.25 / +1.05
Trapping	$F_{TR}$	*trans to cis*	5 mW/15 mW	−3.94 / −4.39
Drag	$F_{D}$	*trans to cis*	110 mV / 190 mV	−1.75 / −1.94
Electroosmotic	$F_{EOF}$	*trans to cis*	110 mV / 190 mV	−1.30 / −1.56
Brownian	$F_{BR}$	Any Directions		1.2 × 10^–3^

## Data Availability

The authors do not have permission to share data.
